# Novel Role of Pin1-Cis P-Tau-ApoE Axis in the Pathogenesis of Preeclampsia and Its Connection with Dementia

**DOI:** 10.3390/biomedicines13010029

**Published:** 2024-12-26

**Authors:** Emmanuel Amabebe, Zheping Huang, Sukanta Jash, Balaji Krishnan, Shibin Cheng, Akitoshi Nakashima, Yitong Li, Zhixong Li, Ruizhi Wang, Ramkumar Menon, Xiao Zhen Zhou, Kun Ping Lu, Surendra Sharma

**Affiliations:** 1 Division of Basic Science and Translational Research, Department of Obstetrics and Gynecology, The University of Texas Medical Branch at Galveston, Galveston, TX 77555, USA; emmamabe@utmb.edu (E.A.); zhehuang@utmb.edu (Z.H.); ra2menon@utmb.edu (R.M.); 2 Department of Molecular Biology, Cell Biology and Biochemistry, Warren Alpert Medical School of Brown University, Providence, RI 02903, USA; sukanta_jash@brown.edu; 3 Mitchell Center for Neurodegenerative Diseases, Department of Neurology, The University of Texas Medical Branch at Galveston, Galveston, TX 77555, USA; bakrishn@utmb.edu; 4 Department of Pediatrics, Warren Alpert Medical School of Brown University, Providence, RI 02903, USA; shibin_cheng@brown.edu; 5 Department of Obstetrics and Gynecology, Faculty of Medicine, University of Toyama, Toyama 930-8555, Japan; akinaka@med.u-toyama.ac.jp; 6 Departments of Biochemistry and Oncology, Schulich School of Medicine and Dentistry, Robarts Research Institute, Western University, London, ON N6A 3K7, Canada; yli5227@uwo.ca (Y.L.); zli3273@uwo.ca (Z.L.); rwang849@uwo.ca (R.W.); xzhouenator@gmail.com (X.Z.Z.); kunping.lu@gmail.com (K.P.L.); 7 Departments of Pathology and Laboratory Medicine, Schulich School of Medicine and Dentistry, Lawson Health Research Institute, Western University, London, ON N6A 3K7, Canada

**Keywords:** preeclampsia, dementia, peptidyl-prolyl cis–trans isomerase, phosphorylated tau, apolipoprotein E, autophagy, tauopathy, Alzheimer’s disease

## Abstract

Preeclampsia (preE) is a severe multisystem hypertensive syndrome of pregnancy associated with ischemia/hypoxia, angiogenic imbalance, apolipoprotein E (ApoE)-mediated dyslipidemia, placental insufficiency, and inflammation at the maternal–fetal interface. Our recent data further suggest that preE is associated with impaired autophagy, vascular dysfunction, and proteinopathy/tauopathy disorder, similar to neurodegenerative diseases such as Alzheimer’s disease (AD), including the presence of the cis stereo-isoform of phosphorylated tau (cis P-tau), amyloid-β, and transthyretin in the placenta and circulation. This review provides an overview of the factors that may lead to the induction and accumulation of cis P-tau-like proteins by focusing on the inactivation of peptidyl-prolyl cis–trans isomerase (Pin1) that catalyzes the cis to trans isomerization of P-tau. We also highlighted the novel role of the Pin1-cis P-tau-ApoE axis in the development of preE, and propagation of cis P-tau-mediated abnormal protein aggregation (tauopathy) from the placenta to cerebral tissues later in life, leading to neurodegenerative conditions. In the case of preE, proteinopathy/tauopathy may interrupt trophoblast differentiation and induce cell death, similar to the events occurring in neurons. These events may eventually damage the endothelium and cause systemic features of disorders such as preE. Despite impressive research and therapeutic advances in both fields of preE and neurodegenerative diseases, further investigation of Pin1-cis P-tau and ApoE-related mechanistic underpinnings may unravel novel therapeutic options, and new transcriptional and proteomic markers. This review will also cover genetic polymorphisms in the ApoE alleles leading to dyslipidemia induction that may regulate the pathways causing preE or dementia-like features in the reproductive age or later in life, respectively.

## 1. Introduction

Preeclampsia (preE) is a severe multisystem hypertensive syndrome of pregnancy [1,2], characterized by sudden-onset hypertension after 20 weeks of gestation and at least one of the following associated complications: proteinuria or maternal organ dysfunction [1]. It entails poor spiral artery remodeling and contributes to fetal growth restriction (FGR) [1]. It can manifest as an early-onset (diagnosed <34 weeks’ gestation) or late-onset (diagnosed ≥34 weeks’ gestation) condition. Mechanistically, preE has been associated with ischemia/hypoxia, angiogenic imbalance, placental insufficiency, and inflammation at the maternal–fetal interface [1,3,4,5,6,7]. preE is a leading cause of maternal and perinatal morbidity and mortality, cesarean section, prematurity, maternal intensive care admissions, and long-term cardiovascular disease in the mother [3,4,5,8,9,10]. About 4 million women are diagnosed with preE worldwide each year, and it is responsible for over 70,000 and 500,000 deaths of women and babies, respectively [11,12]. The COVID-19 pandemic further exposed the vulnerability of infected pregnant women to develop preE at a much higher rate compared to non-infected pregnant women [13,14,15]. Although delivery resolves most of its signs and symptoms, preE can persist after delivery and can occur de novo in the postpartum period in some cases [16]. 

The tubulin associated unit (tau) protein is a microtubule-associated protein (MAP) that coordinates the formation and stabilization of microtubules in axons [17]. In humans, it is encoded by the microtubule-associated tau protein (MAPT) gene on chromosome 17q21.31 [18] that contains 16 exons [19]. Tau facilitates microtubule-associated axonal transport [20] and synaptic plasticity [21]. Tau is also a negative regulator of mRNA translation by binding to ribosomes, which inhibits ribosomal function, reduces protein synthesis, and alters synaptic function [17,22,23,24,25]. At the cellular level, tau is involved in cell signaling, neuronal development and protection, and apoptosis [26]. Tau’s primary neurodegenerative functions include negative regulation of long-term memory and promotion of habituation (a non-associative learning) [17].

Normal tau is phosphorylated at numerous serine/threonine and tyrosine residues to phosphorylated tau (P-tau), which is susceptible to oligomerization, aggregation, and tangle formation [27,28,29]. Notably, phosphorylation of tau on the Thr231-Pro232 motif (P-tau) exists in two distinct cis (pathogenic) and trans (physiologic) conformations [30]. Tau phosphorylation decreases with aging, but increases significantly in Alzheimer’s disease (AD) [31,32,33,34,35] and chronic traumatic encephalopathy (CTE) due to traumatic brain injury (TBI) [36,37,38,39,40]. Following TBI in mice and stress in vitro, neurons acutely produce cis P-tau, which disrupts microtubule networks and mitochondrial transport in the axons, spreads to other neurons, and leads to apoptosis. This process, which is now termed ‘cistauosis’, appears long before other tauopathy [36].

After phosphorylation, tau conformation and function are also regulated by a stress responsive enzyme called peptidyl-prolyl cis–trans isomerase (Pin1) [41]. Pin1 catalyzes the cis to trans isomerization of the pThr231-Pro motif (P-tau) in tau [42], restoring its physiologic microtubule assembly function, and facilitating its dephosphorylation (by protein phosphatase 2A) and degradation (proteolysis) [43]. As seen in AD, TBI, or stroke, Pin1 is inhibited by phosphorylation [44,45], oxidation [36,46], cytoplasmic sequestration [43], or mutations [30,47]. The resultant hyperphosphorylation of tau enhances its resistance to degradation, maintains the pathogenic cis stereo-isoform of P-tau231 (cis P-tau), inhibits its physiologic function, and propagates the neuropathological cistauosis characteristic of neurodegenerative diseases such as AD, CTE after TBI, vascular contribution to cognitive impairment and dementia (VCID) or vascular dementia (VaD) after stroke long before tangle formation [30,36,43,44,48]. Therefore, in a physiologic state, phosphorylation and dephosphorylation of the tau protein are maintained in a dynamic equilibrium [29].

preE is the first model of an extracerebral cis P-tau mediated tauopathy in a young population represented by pregnant women [30,49]. Despite being an extracerebral tauopathy, preE is associated with a risk of cognitive impairment and dementia in mothers [50,51,52,53,54,55] and their offspring [56,57,58] later in life [59,60]. In several large studies of more than 3 million women [50,51,55,61,62,63], those exposed to preE had about 3-fold increased risk of vascular dementia (hazard ratios 1.63–3.46), even before the age of 65 years [55]. Recent observations suggest microchimeric fetal cells and placental exosomes are also important contributing factors to the preE pathology [64,65,66,67,68]. Interestingly, tau is one of the biomarkers for predicting preE complications [69,70], and P-tau is adjudged a better predictor of preE pathology than total tau [41]. However, data on the etiological mediators of preE and its link to dementia are still very limited. Moreover, the question of whether or how P-tau is involved in this association is still largely unknown. It was only recently that Jash et al. [49] reported that cis P-tau is a pivotal circulating etiological mediator of preE, and that cis P-tau stereo-specific monoclonal antibody (cis mAb) could be useful for early diagnosis and treatment of preE [49]. We demonstrated that preE shares etiological similarities with neuro-degenerative diseases such as AD, involving impaired autophagy and proteinopathy/tauopathy disorder [49,71,72,73,74,75]. These observations suggested that preE may have long-term consequences resulting in dementia. We postulate that in preE, there could be inactivation of Pin1 and activation of cis P-tau and other phosphorylated tau proteins. Other biomarkers we detected in preE include transthyretin, amyloid β-42, and TAR DNA-binding protein 43 (TDP-43) (Table 1) [71,72,73,74,75]. 

We also postulate that tauopathy can be regulated by metabolic abnormalities such as lipid dysfunction controlled by apolipoprotein E (ApoE), which is an essential ligand for the uptake and clearance of atherogenic lipoproteins [90,103,104]. ApoE is a 34 kDa plasma glycoprotein expressed in various tissues, and acts as a lipid and cholesterol transporter [90]. It is present in all lipoproteins except low-density lipoprotein (LDL) and plays a crucial role in clearing atherogenic lipoproteins from plasma into cells [90,103,104]. The ApoE gene has three variant alleles: ε2 (E2), ε3 (E3), and ε4 (E4) [90,91]. The polymorphic nature of ApoE is a significant risk factor for neurodegenerative diseases like AD [94] and cardiovascular disease (CVD) [91,105]. Interestingly, ApoE alleles are also linked to the progression of preE [106,107]. Compared to the most common and relatively neutral E3 allele [90,91,92,93], the ApoE4 allele poses the highest risk for AD [92,108,109,110], CVD [91], and preE [106,107,111], by influencing tauopathy, while the ApoE2 allele is thought to offer protection against AD [92,108,109,110] and preE [88,106]. 

Although AD and preE share pathological similarities, it remains unclear if tau aggregates correlate with ApoE in preE [112,113,114]. In AD, histopathological evidence shows a relationship between ApoE and neurofibrillary tangles in neurons [97,98,99,100]. ApoE4 carriers exhibit severe and typical medial and temporal tau spread characteristics of AD (Braak staging system) along with cognitive decline [97,98,99,100]. ApoE4-induced AD-related pathological changes include impaired autophagy and mitophagy, dysregulated endosomal-lysosomal pathways, enlarged endosomes, and reduced mRNA levels of autophagy markers. ApoE4 also leads to increased tau-induced neurodegeneration and enhanced tau phosphorylation, which are associated with increased P-tau and tau tangle-like structures [96]. Mice expressing ApoE4 show the most significant tau-induced neurodegeneration compared to ApoE2, while ApoE knockout mice are protected from ApoE4’s harmful effects [101]. The ApoE3 allele shows some correlation with AD-related tauopathy but generally acts as a neutral allele. Notably, women carrying the ApoE4 allele exhibit more widespread tau pathology in their brain tissues than men [115,116]. Additionally, ApoE4- and ApoE3-associated tauopathy and tau-mediated neurodegeneration in mouse models can be influenced by gut microbiota-derived short-chain fatty acids (SCFAs) [117]. Besides its protective role against amyloid-β (Aβ) deposition in AD [86], ApoE2 is linked to reduced regional tau in all brain regions, while ApoE4 is associated with greater regional tau, mainly in the medial temporal lobe [87]. The impact of ApoE genotypes, particularly ApoE4, on AD-related tauopathy is still debated due to a possible Aβ-ApoE connection and its effect on tauopathy progression. These findings highlight the need for further research into the ApoE-tau axis in the development of preE and AD-like diseases. 

This review provides an overview of the factors that lead to the inactivation of Pin1 and activation of cis P-tau and other phosphorylated tau proteins in preE, how the Pin1-cis P-tau balance is regulated in preE, and whether a dysregulation in this axis could be related to impaired autophagy, cistauosis, and development of preE. How the Pin1-cis P-tau axis mediates the link between preE and dementia, diagnostic and therapeutic potentials of cis mAb, and avenues for future research are also discussed. We will also discuss how ApoE and tauopathy may converge on programming preE and AD-like health risks in women. 

## 2. Inactivation of Pin1 and Activation of Cis P-Tau and Other Phosphorylated Tau Proteins in Preeclampsia

**Figure 1:**
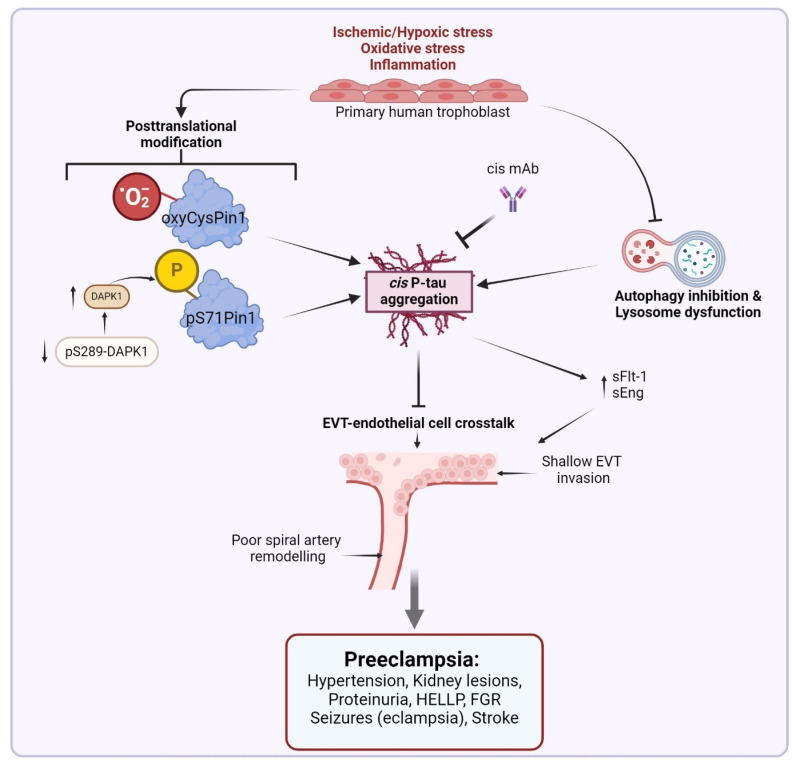
I. Inactivation of the Pin1-cisP-tau axis induces preeclampsia features. Exposure of trophoblast cells to hypoxic stress or serum from preeclampsia patients inhibits Pin1 by oxidation or phosphorylation, thereby facilitating cis P-tau aggregation. The protein aggregation may also be secondary to impaired autophagic clearance induced by hypoxia or preeclampsia serum. Elevated cis P-tau aggregates inhibit the interaction between EVT and spiral artery endothelium, leading to shallow EVT invasion and poor vascular remodeling that precipitate preeclampsia features. Hypoxia also induces the production of sFlt-1 and sEng in trophoblasts mediated by or independent of cis P-tau aggregation. sFlt-1 and sEng can cause vascular endothelial dysfunction that ultimately results in the preeclampsia syndrome. Importantly, cis mAb inhibits the accumulation of cis P-tau, thereby re-storing normal placental development and function. DAPK1, death associated protein kinase 1; EVT, extravillous trophoblast; FGR, fetal growth restriction; HELLP, hemolysis, elevated liver enzymes, and low platelet count; Pin1, peptidyl-prolyl cis–trans isomerase; pS289-DAPK1, DAPK1 phosphorylated at serine residue 289; sEng, soluble endoglin; sFlt-1, fms-like tyrosine kinase 1. Created with BioRender.com.

In physiological conditions, P-tau is mostly maintained in the trans P-tau conformation. However, in stressful conditions, cis P-tau is induced, and Pin1 is inactivated and fails to catalyze isomerization of the pathogenic cis P-tau to the physiologic trans P-tau [30]. Inactivation of Pin1, as seen in neurodegenerative conditions and preE, can lead to excessive accumulation of cis P-tau (Figure 1), which dissociates from microtubules, becomes resistant to dephosphorylation and degradation, oligomerizes, and ultimately aggregates into neurofibrillary tangles, leading to disintegration of the microtubules [30,118]. 

In both in vivo and in vitro studies, exposure of primary human trophoblast to hypoxia and serum from preE patients induces cis P-tau and anti-angiogenic factors such as soluble fms-like tyrosine kinase 1 (sFlt-1) and soluble endoglin (sEng) [49] (Figure 1). Elevated cis P-tau was observed in the placenta and blood samples of both early- and late-onset preE patients, as well as in primary human trophoblast exposed to preE patient sera or hypoxia. These observations were attributed to Pin1 inactivation. Moreover, preE placental syncytiotrophoblast (STB) showed high cis P-tau colocalized with other hyperphosphorylated tau isoforms, including pT231-tau, AT8-tau, pS396, and T22 oligomeric-tau, but not AT100-tau associated with mature tangles [49]. 

Similar to AD, TBI/CTE, and stroke/VaD, our group demonstrated a preE-associated Pin1 inactivation by cysteine-113 (Cys113) oxidation [36,46], and Ser71 phosphorylation by death associated protein kinase 1 (DAPK1) [44,45,119,120], and concomitant induction and cytoplasmic sequestration of cis P-tau and protein aggregation [49]. Oxidized Oxy-Cys113 Pin1 and phosphorylated pS71-Pin1 were more abundant in placental tissues of preE patients irrespective of preE onset compared to normotensive controls, consistent with induction and cytoplasmic sequestration of cis P-tau [49]. Abundant Oxy-Cys113 Pin1 and pS71-Pin1 were observed in STB and cytotrophoblast (CTB) in early preE placental sections by immunohistochemical staining compared to the very weak and sparse staining observed in control samples. However, there was no significant change in total Pin1 in preE vs. control placenta [49]. Our findings corroborate and extend previous reports of increased circulating DAPK1 in women destined to develop preE and those with confirmed preterm preE [121]. Similarly, placental DAPK1 mRNA and protein expression were increased in women with preE [121]. DAPK1 appears to promote apoptosis and autophagy [121,122]. However, when we exposed primary human trophoblasts to hypoxia, we observed that pS289-DAPK1, which inhibits DAPK1 cellular function [123], was reduced, while DAPK1 was significantly elevated [49], a possible mechanism for Pin1 post-translational modification and inactivation [43,44,45,46] (Figure 1). Furthermore, administration of Sulfopin, which targets the Cys113 and ATO-binding pocket in the active site of Pin1, inhibited the enzyme and caused accumulation of cis P-tau and protein aggregates in primary trophoblast cells as well as in pregnant humanized tau (hTau) mice. In hTau mice, cistauosis and protein aggregation were accompanied by a preE phenotype, including proteinuria, increased sFlt-1 and sEng production, and fetal growth restriction (FGR). These were accompanied by elevated blood pressure and decreased uterine and umbilical arterial systolic velocities on gestational day 16.5 [49]. When cis P-tau was depleted from the sera of preE patients by cis mAb, the ability of cis P-tau to alter trophoblast invasion and endovascular activity to cause preE-like pathological and clinical features in the hTau mice was diminished (Figure 1) [49]. Therefore, we posited that in preE there is inactivation of Pin1, which leads to the accumulation of cis P-tau that could be a pivotal early driver and useful biomarker for the syndrome. Importantly, cis P-tau can be targeted by stereo-specific mAb for early diagnosis and treatment [49]. 

Notably, cis P-tau is an established early mediator and biomarker of early-stage AD, TBI, and stroke, which are confirmed risk factors for dementia [37,38,39,124,125], with a cis P-tau mAb currently in clinical trials [41,126,127]. Moreover, mothers and their offspring who experienced preE are at risk of dementia later in life [50,51,54,55,56,57,58,59,60]. These results suggest a pathomechanical link between preE and dementia, with cis P-tau playing an important role. This hypothesis is on the premise that preeclamptic women demonstrated more than 2-fold higher plasma tau compared to women with normotensive pregnancies. The preeclamptic women with neurologic complications, including eclampsia (seizures), cortical blindness, and hemorrhagic stroke, had about 3-fold greater tau and glial fibrillary acidic protein (GFAP) compared to preeclamptic women without neurologic complications or pulmonary edema, hemolysis, elevated liver enzymes, and low platelet count (HELLP) [69]. The women with HELLP had more than 4-fold higher tau and greater than 1.5-fold higher neurofilament light (NfL) and GFAP compared to preeclamptic women without neurologic complications, pulmonary edema, or HELLP. Plasma concentrations of tau and GFAP were also elevated in women with severe neurologic complications compared to those with eclampsia only [69]. Consequently, the authors concluded that tau, NfL, and GFAP were promising biomarkers for the diagnosis and prediction of cerebral complications of preE [69]. This is logical as tau and NfL are increased in women destined to develop preE, even after the diagnosis [128,129,130,131,132], as well as when there is axonal injury [133,134,135,136,137]. GFAP also increases when the glial cells are involved [69,134]. Mechanistically, the link between severe preE, eclampsia, and cerebral injury could be a decrease in cerebral blood flow (CBF) and increased blood–brain barrier (BBB) permeability, leading to cerebral edema and neuroinflammation [138]. 

Women who experience preE are prone to acute neurologic complications, such as seizures (eclampsia), cerebral edema, intracerebral hemorrhage [138], and long-term complications, including dementia, epilepsy, and stroke [50,139,140]. Although Bergman et al. [69] did not exclusively implicate cis P-tau as the tau epitope increased in the preE patients with severe neurologic complications, the findings from our studies [49] lend credence to the plausible involvement of the Pin1-cis P-tau axis. We postulate that in severe preE that could lead to neurologic complications such as dementia, the Pin1-cis P-tau axis is disrupted, leading to increased cis P-tau aggregation in placental tissues and cistauosis. This is also supported by the observation that the addition of non-phosphorylated tau to the biomarker panel did not improve the differentiation of AD- and non-AD-associated dementias [136]. 

## 3. Impaired Autophagy, Cistauosis, and Development of Preeclampsia

**Figure 2:**
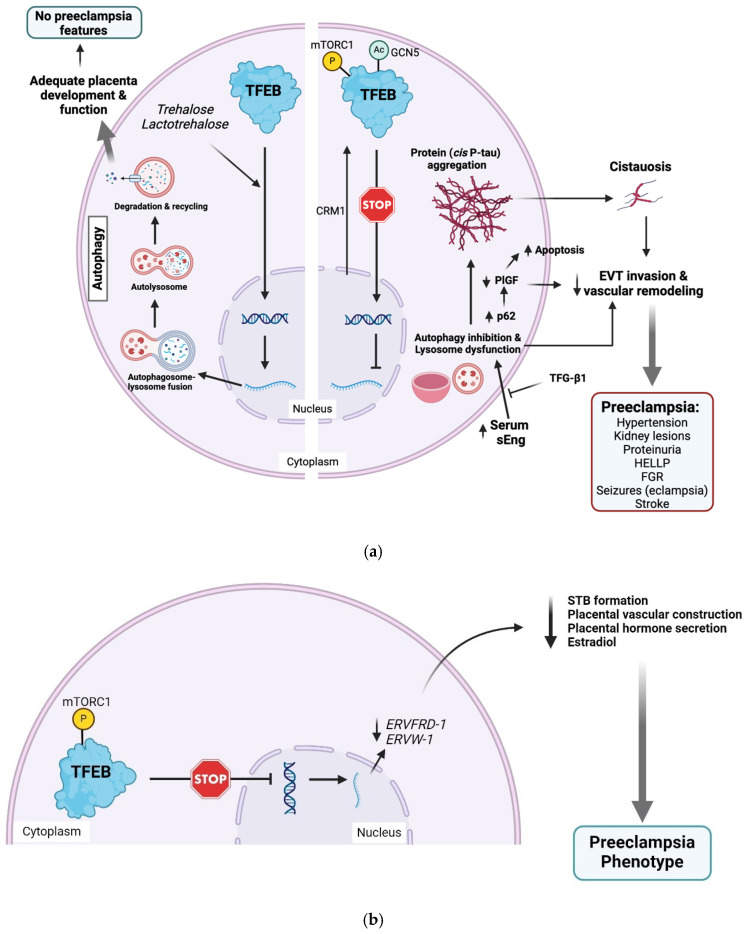
Transcription factor EB mediates placental development. (**a**) TFEB-mediated autophagy–lysosomal dysfunction contributes to protein (cis P-tau) aggregation associated with preeclampsia. Inhibition of nuclear translation or transcriptional activity of TFEB inhibits autophagy and disrupt lysosomal function through reduced protein expression and function. This leads to increased p62 and accumulation of toxic protein aggregates, including cis P-tau, which can leak into circulation (cistauosis). Increased accumulation of p62 in trophoblast cells leads to reduced placental growth factor (PlGF). Moreover, increased serum sEng enhances lysosomal dysfunction. The resultant effect of all these alterations is increased apoptosis, reduced EVT invasion, and inadequate spiral artery remodeling, ultimately inducing preeclampsia features. Phosphorylation (by mTORC1) and acetylation (by GCN5) of TFEB inhibit its nuclear translocation and transcriptional activity, respectively. TFEB nuclear export is also promoted by activated chromosome region maintenance 1 (CRM1). Trehalose is a mTORC1-independent autophagy promoter. Trehalose and lactotrehalose promote autophagy by increasing the nuclear translocation of TFEB. (**b**) Autophagy-independent TFEB-mediated maintenance of placental function. Deficiency or reduced nuclear translocation of TFEB impairs syncytiotrophoblast (STB) formation, placental vascular construction, and hormone production, including estradiol through reduced activation of the fusogens *ERVFRD-1* and *ERVW-1*. These factors can be independently or in synergy with dysfunctional autophagy–lysosomal biogenesis machinery to induce features of preeclampsia. *CRM1*, chromosome region maintenance 1; *ERVFRD-1*, endogenous retrovirus group FRD member 1 (Syncytin-2); *ERVW-1*, endogenous retrovirus group W member 1 (Syncytin-1); *FGR*, fetal growth restriction; *GCN5*, general control non-repressed protein 5; *HELLP*, hemolysis, elevated liver enzymes, and low platelet count; *mTORC1*, mechanistic target of rapamycin complex 1; *TFEB*, transcription factor of the E box. Created with BioRender.com.

Autophagy is a programmed lysosomal degradation pathway utilized by eukaryotic cells to clear long-lived proteins, protein aggregates, damaged organelles, and harmful substances [141,142,143]. Autophagy protects the placenta against stress and harmful substances, including pathogens [143,144,145,146,147,148,149,150]. Recently, our group developed a novel blood diagnostic test for preE and AD employing an autophagy-deficient human trophoblast cellular model combined with a fluorescent dye with specific binding affinity for protein aggregates [72]. The concept emanated from our observations that suggested that preE is associated with impaired autophagy and inefficient lysosomal biogenesis machinery in the placenta [151,152]. We observed accumulation of protein aggregates, impaired autophagy, and transcriptional factor of the E box (TFEB)-mediated defective lysosomal biogenesis in the placenta of preE patients (Figure 2) [151,152,153]. As placental autophagy failure is associated with the risk of preE via protein aggregates comprising of transthyretin, amyloid-β42 [72], and now cis P-tau in preE patients’ sera [49], we postulate that impaired autophagy is responsible for the accumulation of protein aggregates especially cis P-tau in the circulation (cistauosis) and placenta of preE patients.

Using trophoblast-specific conditional Atg7 knockout (Atg7-cKO) mice model [154], autophagy was shown to play a pivotal role in trophoblast invasion and vascular remodeling required for normal placentation in extravillous trophoblasts (EVTs) [155,156,157]. Autophagy deficiency mediates reduced placental growth factor (PlGF) mRNA and abnormal placentation, which is a feature of preeclamptic placentas [154]. Autophagy inhibition evidenced by increased expression of the autophagy substrate, SQSTM1/p62, is observed in EVTs of preeclamptic placentas [158,159]. In trophoblast-specific Atg7-cKO mice, accumulation of p62 in trophoblast cells led to reduced PlGF, increased apoptosis, shallow endovascular trophoblast invasion, and inadequate vascular remodeling [154]. sEng, a TGF-β1 inhibitor, in serum of preeclamptic patients also suppressed trophoblast invasion and vascular remodeling by inhibiting autophagy in EVT cell lines. In this regard, the administration of TGF-β1 produced a reverse effect (Figure 2) [155]. 

We have also shown increased mammalian/mechanistic target of rapamycin com-plex 1 (mTORC1) activity, reduced nuclear translocation of TFEB, and decreased lysosomal protein expression and function in autophagy-deficient human EVT [152]. These features, including reduced TFEB expression and protein aggregation, were reproduced in EVTs exposed to sera from preE patients, as well as in Atg7-cKO mice [152], as previously reported [154]. TFEB is a downstream transcriptional factor of mTORC1 and the master regulator of the autophagy–lysosomal pathway [160,161,162]. Activation of mTORC1 dysregulates lysosomal biogenesis and inhibits autophagy (Figure 2a) [163,164,165] through phosphorylation-dependent inhibition of TFEB, ULK1/2, and the VPS34 complex [166]. mTORC1 is upregulated, while beclin-1 and light chain protein 3 (LC3-II) are downregulated in placental tissues of preE patients [167]. Downregulation of TFEB is accompanied by increased p62 in syncytial BeWo cells [161]. TFEB also mediates trophoblast syncytialization and placenta hormone production [168] independent of the autophagy–lysosomal pathway [160], albeit syncytialization in BeWo cells required autophagy activation [147,169]. Deficiency of TFEB significantly impairs syncytiotrophoblast (STB) formation in human trophoblast [168] and placental organoids [160]. TFEB promoted STB formation by direct transcriptional activation of the fusogen ERVFRD-1 [160] and ERVW-1 [168] in human trophoblast (Figure 2b). By contrast, STB formation and placental vascular construction [160] as well as secretion of placental hormones and estradiol [168] are compromised with systemic or trophoblast-specific deletion of the TFEB gene. 

Recently, we demonstrated that preE-associated impaired autophagy and proteinopathy could be reversed by non-mammalian, natural disaccharide trehalose and its lacto analog lactotrehalose [75]. In the form of a ‘sweet relief’, trehalose and lactotrehalose restored autophagy, inhibited the toxic aggregation of proteins, and restored the ultrastructural features of autophagosomes and autolysosomes in primary human trophoblasts [75]. The disaccharides also had a similar effect on the placenta of humanized mouse models, normalizing the preE-associated transcriptome profile and inhibiting the onset of preE-like features. The sugars restored the autophagy–lysosomal biogenesis machinery by enhancing the nuclear translocation of TFEB (Figure 2a) [75]. Trehalose is an mTOR-independent autophagy promoter and currently a drug of choice for treating several neurodegenerative diseases including AD, Parkinson’s disease, amyotrophic lateral sclerosis, Huntington’s disease, and TBI characterized by impaired autophagy and misfolded proteins [170,171,172,173,174,175,176,177,178]. Therefore, our results show more evidence to support a pathological link between preE and neurodegenerative diseases that can cause dementia, as well as a promising therapeutic intervention for this severe pregnancy complication with lifelong sequelae. 

In another example of TFEB-mediated impaired autophagy and proteinopathy, cadmium-induced acute kidney injury (AKI), it was revealed that TFEB phosphorylation, nuclear export, and reduced transcriptional activity induced by acetylation independently suppressed TFEB activity leading to autophagy-lysosome dysfunction (Figure 2a) [179]. Importantly, inhibition of chromosome region maintenance 1 (CRM1, which enhances TFEB nuclear export) or general control non-repressed protein 5 (GCN5, which mediates TFEB acetylation) alleviated cadmium-induced AKI by enhancing TFEB activity [179]. As we demonstrated with trehalose, if these mechanisms are replicated in placental or other gestational tissues in relation to preE, the inhibitors of CRM1 (leutomycin B), GCN5 (butyrolactone 3) [179], or mTORC1 (rapamycin and its analogs) [180] could be exploited as therapeutics to alleviate preE due to impaired autophagy. Taken together, activation of mTORC1 and/or deficiency of TFEB mediates poor placentation that precedes preE syndrome through impaired autophagy (including defective lysosomal biogenesis), and syncytialization. The downstream effect is accumulation of protein aggregates, which could include cis P-tau (cistauosis) augmented by hypoxia induced by poor spiral artery remodeling due to shallow EVT endovascular invasion (Figure 2a). However, further studies are required to establish a direct link between deficiency of TFEB and cistauosis; and whether trehalose can directly/specifically inhibit cis P-tau aggregation or enhance the other preE-preventing but autophagy-independent functions of TFEB (Figure 2b). Of all human tissues, STB has the largest deposits of TFEB [168] and should be a suitable site for such investigation. As seen in acute kidney injury, the relationship between environmental and occupational exposure to harmful substances like cadmium, and TFEB inhibition and cis P-tau aggregation requires investigation. These investigations are necessary because there are several arguments that autophagy is hyperactivated [165,181,182,183,184,185] instead of deficient in preE even though they do not explain the presence of protein aggregates in serum and placenta from preE patients. In addition, placental autophagy can be hijacked by stress-related events and/or sterile inflammation to elude host immune clearance [141,150,186]. 

## 4. Cis P-Tau Disrupts Endovascular Endothelial Cells-Trophoblast Crosstalk

Optimal interaction between endothelial and trophoblast cells is required for spiral artery remodeling and adequate placental development (Figure 1) [1,187,188]. Sera from preE patients disrupt endovascular interaction between first trimester EVTs (HTR8) with invasive properties (VEGF receptor 2 kinase activity) and primary human umbilical vein endothelial cells (HUVECs) [73,158,189]. Following this observation, it was important to determine the underpinning mechanism(s). Like autophagy inhibition [152,154,155,156,157,158], elevated cis P-tau disrupts the endovascular crosstalk between EVTs and endothelial cells necessary for optimal placentation [49]. High placental expression of cis P-tau induced by hypoxia appears to promote a toxic tau seed (spread) between trophoblast cells and abolish their migration and invasive capability [49]. This action of cis P-tau is also seen in AD, TBI/CTE, and VCID [36,44]. 

**Figure 3:**
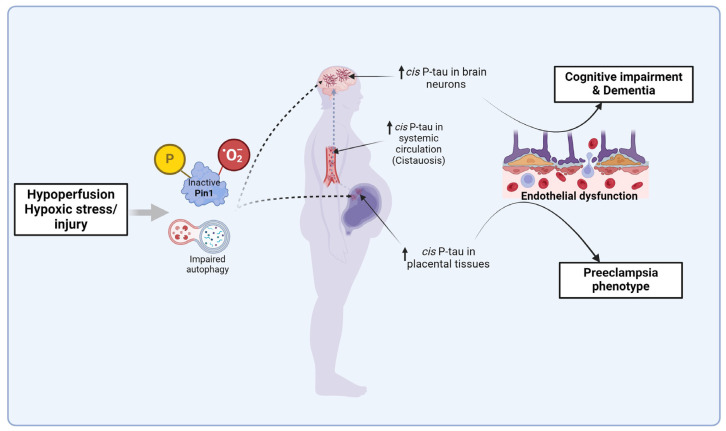
The mechanistic link between preeclampsia and dementia. Both preeclampsia and dementia are mediated by *endothelial dysfunction* induced by the accumulation and spread of cis P-tau aggregates in placental and brain tissues, respectively. cis P-tau accumulates due to inactivated Pin1 and impaired autophagy and lysosomal dysfunction induced by hypoxia secondary to hypoperfusion. Cistauosis due to preeclampsia-related leakage of accumulated placental cis P-tau into systemic circulation can also deliver cis P-tau to brain neurons in the long run, leading to cognitive impairment and dementia. *Pin1*, peptidyl-prolyl cis–trans isomerase; *P-tau*, phosphorylated tau. Created with BioRender.com.

Neurons produce high cis P-tau following TBI in mice and humans, and after exposure to stress (hypoxia or serum starvation) in vitro [36]. Elevated cis P-tau disrupts axonal microtubule networks and mitochondrial transport and spreads to other neurons (as we observed in trophoblast cells) [49], leading to apoptosis [36]. Treating TBI mice with cis mAb inhibits cistauosis, prevents tauopathy development and spread of cis P-tau to other neurons, and restores TBI-associated consequent structural and functional abnormalities seen even in humans [36,190]. Similarly, treating primary human trophoblasts exposed to hypoxia with cis mAb restored their migration and invasive properties required for optimal endovascular crosstalk between EVTs and endothelial cells, and placentation [49]. cis mAb also inhibited disruption of the endothelial cell–trophoblast interaction by preE patient serum [49,189]. Furthermore, the cis P-tau induction in the injured/stressed neurons was significantly associated with Pin1 inactivation by Cys113 oxidation (induced by hypoxia), S71 phosphorylation (induced by TBI), and Pin1 downregulation (induced by serum starvation) [36]. We reproduced this observation in primary human trophoblast exposed to Sulfopin in a time/dose-dependent manner [49]. Sulfopin inhibited Pin1, induced cis P-tau protein aggregation, and appeared to stimulate FLT-1 alternative splicing in a time-dependent (but less so in a dose-dependent) manner [49]. Meanwhile, hypoxia induces the production of sFlt-1 and sEng as well as transcription of alternatively spliced sFlt-1 variants in primary human trophoblasts mediated by cis P-tau aggregation. However, cis mAb blocked the production of these anti-angiogenic factors and reversed transcription of alternatively spliced sFlt-1 variants but had no effect on Eng mRNA expression [49]. Taken together, in brain neurons and placental trophoblasts, hypoxic stress/injury inactivates Pin-1, which induces cis P-tau aggregation that can spread between the cells and disrupt trophoblast–endothelial interactive functions, leading to dementia and preE features, respectively. This indicates that preE and dementia share a common pathophysiological pathway (Figure 3), and could benefit from the same biomarker and therapeutic intervention.

Another example of Pin1 inactivation mediating endothelial dysfunction has been shown in VCID or vascular dementia (VaD) [124,125]. Unlike AD, VCID is characterized by robust cis P-tau but no hyperphosphorylated tau neurofibrillary tangles in both human and mouse models [44]. VCID-associated induction of cis P-tau was attributed to the inactivation of Pin1 by DAPK1-induced phosphorylation [44], as we demonstrated in placental tissues [49]. Moreover, other studies have also reported increased levels of several tau epitopes in VCID patients and mouse models with cerebral hypoperfusion [41,191,192,193]. Targeting such tau epitopes prevents and ameliorates neuroinflammation, cognitive impairment, and memory loss in mice with cerebral hypoperfusion [44,191]. VCID is precipitated by white matter lesions induced by oxidative stress-induced endothelial dysfunction that disrupts the trophic interaction between endothelial cells, oligodendrocytes (that produce myelin), and neurons. Endothelial dysfunction causes decreased cerebral blood flow (CBF) in the white matter, which receives limited blood supply, and increased permeability of the blood–brain barrier (BBB). Hypoperfusion and increased permeability of the BBB leads to tissue hypoxia and extravasation of plasma proteins. This exacerbates the oxidative stress and the resultant tissue edema from increased BBB permeability compress the blood vessels to further reduce CBF, thereby creating a harmful positive feedback cycle [30]. Apart from the impending deleterious inflammation, reduced CBF inhibits Pin1 by activating its inhibitory kinase DAPK1, and induces cis P-tau before the appearance of VCID-like features. These pathological processes are prevented and mitigated by cis mAb, Pin1 overexpression, or DAPK1 knockout, as shown in mice [44]. From the above instances, we can infer that dysregulation of the Pin1-cis P-tau balance leads to elevated cis P-tau, which disrupt endothelial function, and, in preE, disrupts endovascular crosstalk between EVTs and endothelial cells. This negatively impacts placentation and precipitates preE features (Figure 3). This supports the use of P-tau or tau species in predicting the complications of preE, including the cognitive ability of preE patients [41,69,70]. 

## 5. Tauopathy and ApoE-Mediated Abnormal Lipid Metabolism

**Figure 4:**
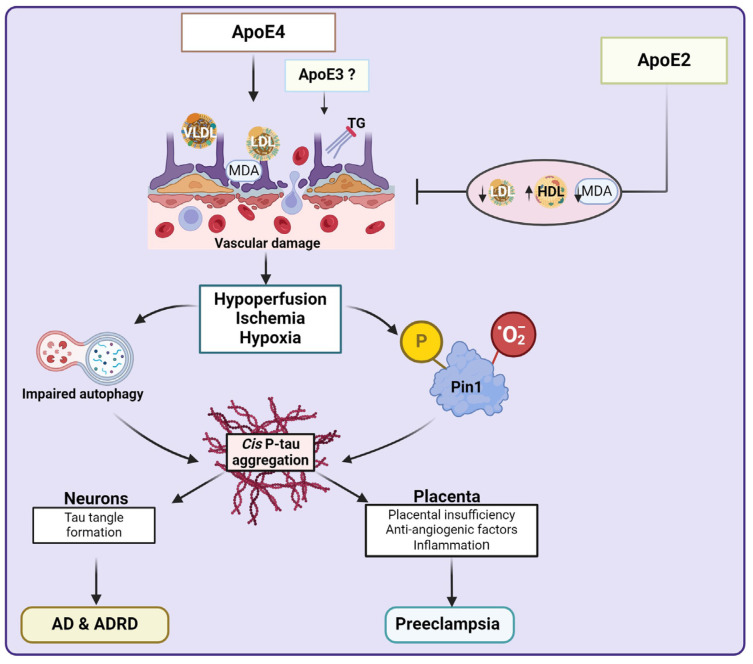
Mechanisms of ApoE-mediated dyslipidemia, vascular dysfunction, and tauopathy. The accumulation of cis P-tau aggregates seen in brain and placental tissues of Alzheimer’s disease (AD) and preeclampsia patients, respectively, could be induced by hypoxia-associated impaired autophagy and Pin1 activation precipitated by vascular damage due to dyslipidemia. This sequence of pathologic events is seen in ApoE4 carriers, while ApoE2 seems to confer some protection by preventing vascular damage through the reduction of oxidative stress marker (MDA), LDL and increasing HDL. *ADRD*, Alzheimer’s disease-related disorders; *ApoE*, apolipoprotein E; *HDL*, high-density lipoprotein; *LDL*, low-density lipoprotein; *MDA*, malondialdehyde; *P-tau*, phosphorylated tau; *Pin1*, peptidyl-prolyl cis–trans isomerase; *VLDL*, very low-density lipoprotein. Created with BioRender.com.

ApoE mediates the binding of lipoproteins or lipid complexes in the plasma or interstitial fluids to specific cell-surface receptors, including LDL receptor, low-density lipoprotein-related protein, and very-low-density lipoprotein (VLDL) receptor [90]. Despite sharing similarities with AD pathology, it is not clear whether tau aggregates correlate with ApoE in the context of preE. Still, there is histopathologic evidence of a relationship between ApoE and the formation of intracellular neurofibrillary tangles (NFT) in AD brains [112,113,114]. In collaboration with phosphorylated neurofilaments and P-tau, carboxyl-truncated fragments of ApoE induce NFT-like structures in AD brains, cultured neurons [112], and mouse neuroblastoma cells [194]. This AD-related assemblage was not observed in non-neuronal cells [112], even in the presence of neurofilaments or tau [194], and was more profound with ApoE4 than ApoE3 [112]. ApoE4 carriers display severe and typical medial and temporal spread of tau characteristic of AD (Braak staging pattern) along with cognitive decline [97,98,99,100]. At the same time, non-ApoE4 carriers exhibit a non-Braak pattern of tau uptake plausibly mediated by amyloid-β deposition [195,196,197]. ApoE4-induces several AD-related autophagy anomalies and increased tau-induced neurodegeneration, enhanced tau phosphorylation, and increased cleavage of C-terminal truncated ApoE fragments that are associated with increased P-tau and NFT-like structures (Figure 4) [96]. Interestingly, women carrying ApoE4 show a more widespread brain tau pathology [108,115,116,198] and faster decline in CBF [199] than males, suggesting an interactive rather than independent effect of ApoE and sex on cerebral tau pathology [198] and blood flow [199].

Transgenic mice expressing ApoE4 exhibit the most severe tau-induced neurodegeneration, whereas ApoE2 and ApoE3 result in less severe effects, and ApoE knockout mice are protected from these devastating effects [101]. In other mechanistic studies in the context of tauopathy, the ApoE-mediated neurodegeneration could be achieved by modulating microglial activation instead of tau-induced direct neurotoxicity [200]. Microglia-mediated inflammation drives tau pathology to the extent that the progression of tau pathology was inhibited in the absence of microglia [200]. This raises the question of whether the relationship between ApoE and tauopathy is direct or modulated by other factors including amyloidosis [108,115,201,202]. 

The vascular endothelial growth factor (VEGF) family, which comprises homodimeric ligands (VEGFA, VEGFB, VEGFC, VEGFD, and placental growth factor (PlGF)), receptors (Flt-1, KDR, and Flt-4), and co-receptors (NRP1 and NRP2), each with several isoforms [203,204], is involved in angiogenesis, neurogenesis, and neuronal survival [205,206,207]. Like in serum and the placenta of preE patients with impaired placental angiogenesis [208,209,210,211], decreased VEGFA (the most well-studied ligand of the family) in serum and cerebrospinal fluid (CSF) is associated with a greater risk of AD and cognitive decline [203,212,213], although not without contrasting opinions [214,215]. However, VEGFA is particularly protective in individuals at the highest risk of AD and cognitive impairment [102] because elevated CSF VEGFA is associated with delayed hippocampal atrophy and cognitive decline in AD patients [213]. Moreover, the neuro-protective role of VEGFA has been supported by cognitive improvement in AD mice models treated with VEGFA [216,217]. Treatment with VEGFA also resulted in recovery from behavioral impairments in humanized ApoE4 mice [218]. Notably, both VEGFA and its receptor VEGFR-1 (Flt-1) have multiple spliced variants that are either pro-angiogenic or anti-angiogenic [219,220,221,222]. Flt-1, KDR (VEGFR-2), and NRP1 spliced variants can exist as transmembrane receptors that signal intracellular receptor tyrosine kinase (RTK) cascades or truncated soluble receptors such as sFlt-1 that scavenges agonists of Flt-1 [203,223,224] leading to the multisystem endothelial dysfunction characteristic of preE [225,226,227]. VEGFA binds to NRP1 to promote angiogenesis [203,228,229,230], vascular permeability [227,231] and prevent ischemia [102]. However, this action may be detrimental in the brain tissues of ApoE4 carriers that are especially susceptible to increased leakage of the BBB resulting in ischemia, neurodegeneration, and cognitive impairment (Figure 4) [232,233]. On the other hand, this plausibly VEG-FA/NRPI-mediated angiogenic or endothelial cell remodeling process is beneficial in ApoE4 non-carriers [102]. It highlights another harmful vascular effect of ApoE4 that could be investigated in placental tissues in relation to preE.

Vascular endothelial injury/dysfunction is central to the pathogenesis of preE and dementia (Figure 4) [234]. ApoE is one of the most debated genes related to preE complications [106,107,235]. There is a correlation between abnormal lipid metabolism (dyslipidemia) and endothelial dysfunction [236,237] that may precipitate preE. Reduced ApoE (perhaps the protective isoform) is associated with low high-density lipo-protein (HDL), which impairs reverse cholesterol transport and may increase the risk of vascular damage [238]. Even without a significant change in serum lipid levels, absence/deficiency of the protective ApoE as in ApoE knockout mice [239] could produce preE features, including elevated Toll-like receptor 4 (TLR4) and sFlt-1 expression, as well as high systolic blood pressure, proteinuria, thickening and edema of glomerular filtration membrane, and capillary thrombosis [239]. Significant edema and necrosis of placental villus stroma is also observed [239]. Therefore, the absence of the protective ApoE could induce PE-associated endothelial dysfunction through dysregulated lipid metabolism and the release of anti-angiogenic factors. This hypothesis is partly supported by the lower frequency of the protective ApoE2 in some women with severe preE compared to normotensive controls [88,106]. ApoE2 is reported to be protective against severe preE through its antioxidant capacity, reducing serum levels of malondialdehyde (MDA), a marker for oxidative stress that alters endothelial function [88,89]. The MDA, which was highest in ApoE4 carriers compared to the other two isoforms, correlated negatively with HDL-cholesterol (Figure 4) but positively with diastolic blood pressure [88]. preE patients with ApoE2 also exhibit lower LDL-C compared to preE patients carrying ApoE4 and ApoE3 [104]. Taken together, perhaps the preE features observed in ApoE knockout mice [239] were as a result of loss of the protective (antioxidant and LDL-C lowering) actions of ApoE2 (Figure 4). 

Importantly, the placenta is involved in cholesterol metabolism at the maternal–fetal interface by secreting anti-atherogenic apolipoproteins like ApoE s. This was reported as higher concentrations of ApoE were found in maternal than fetal serum, and primary trophoblast cells were found to secrete significantly higher amounts of ApoE and ApoA1 than hepatocytes [240]. Pregnant women with ApoE4 have reduced ApoA1 and HDL compared to ApoE2 and ApoE3-positive women [104]. preE mothers exhibit high plasma levels of total cholesterol, LDL-cholesterol, and TG [241,242,243,244], and those of them with ApoE4 deliver earlier neonates with lower birth weight compared to homozygotic ApoE3 preE mothers [111]. Nonetheless, the association of preE with ApoE polymorphism is still debatable and warrants extensive research to validate this concept.

We hypothesize that carrying ApoE2 or ApoE3 is safer than not having ApoE or carrying ApoE4. In brain tissues, ApoE4 induces dyslipidemia leading to vascular dysfunction, reduced CBF, ischemia, hypoxia, inhibited autophagy, and may inactivate Pin1 (Figure 4). This can eventually lead to accumulation of toxic P-tau aggregates that are associated with AD and dementia. Though not resolved in placental tissues yet, ApoE4 or absence of ApoE can induce similar vascular dysfunction-hypoxia-mediated tauopathy associated with preE (Figure 4). The ApoE polymorphic link between preE and AD may be mediated by the protective action of ApoE2 common to both conditions, albeit driven by different mechanisms. Further investigation is warranted to clarify whether ApoE2 is associated with reduced aggregation of P-tau in serum or placenta of preE patients. In this regard, the actions of the different proteins associated with preE and dementia are summarized in Table 1.

## 6. Discussion and Future Perspectives

preE is a non-neuronal instance of inactivation of Pin1 that is coupled with impaired autophagy, probably due to deficiency of TFEB and increased sEng, ultimately leading to cis P-tau aggregation in placental tissues and systemic circulation (cistauosis). Cistauosis facilitates endothelial damage in placental and brain tissues, and appears to be the mediator of the association between preE and risk of cognitive impairment and dementia in mothers and their offspring later in life [50,51,52,54,55,56,57,58,59,60]. This is also plausible because cistauosis appears long before other tauopathies [36], and cis P-tau is an early mediator and biomarker of early stage and pre-clinical AD, TBI, and stroke/VaD/VCID that are associated with dementia [37,38,39,48,124,125,245,246,247,248]. Because preE can occur post-partum, in the absence of a placenta, indicating a persistent systemic dysfunction independent of the presence of the placenta [81], we hypothesize that a cistauosis-mediated tauopathy may be involved. 

There is growing evidence that preE is an example of a non-neuronal disease-associated Pin1 inactivation by oxidation and phosphorylation, and concomitant intracellular accumulation of pathogenic cis P-tau aggregates that could leak into the circulation after attaining a specific critical threshold [49], in line with general protein misfolding, aggregation and aberrant/incomplete degradation (proteolysis) mechanisms [81,249,250,251,252]. The toxic cis P-tau inhibits trophoblast endovascular invasion of the decidua [49,188]. Hence, spiral artery remodeling is compromised [188], leading to poor blood flow to the intervillous space, placental ischemia and hypoxia, and STB stress (stage 1 of preE), which precipitates the maternal clinical features of preE (stage 2) [1,81,253,254]. STB stress (stage 1) is characterized by endoplasmic reticulum (ER) stress, oxidative stress, mitochondrial damage, dysregulated metabolism, and apoptosis [253,255,256]. The stressed STB releases pro-inflammatory cytokines, reactive oxygen species, sFlt-1, sEng, cell-free fetal DNA, and extracellular vesicles (EVs) with specific cargo that can induce the pathobiological changes in other cells by paracrine signaling [1,253,254,257,258,259], and plausibly misfolded (aggregated) proteins including cis P-tau [81] into the maternal circulation. These factors disrupt maternal endothelial function and promote systemic multi-end-organ damage that involves reduced vasodilation, generalized excessive vascular inflammation, and thrombosis (stage 2) [81,253,260,261,262]. The resultant effects include hypertension, liver and renal impairment, proteinuria, pulmonary edema, thrombocytopenia and coagulopathy [1,253], visual disturbances, persistent headache, stroke, and eclampsia (seizures) [263]. This cascade of events and the resultant phenotype are similar to the pathophysiology of VCID, including AD, where tissue hypoxia and oxidative stress induced by reduced CBF and increased BBB permeability stimulate toxic cis P-tau and other tau epitopes aggregation [41,44,191,192,193] and neuroinflammation that damage the neurovascular units leading to progressive neuronal axon, myelin, and endothelial cell degeneration [124,264,265,266]. A similar vascular dysfunction-hypoxia-induced tauopathy and tau-mediated toxicity is seen in carriers of certain ApoE isoforms (especially ApoE4) that cause dyslipidemia and impaired autophagy in the brain [94,96,101,102] and possibly in the placenta [239,240,267]. Although the cells exist at different body sites (placental tissues vs. brain neurons), the shared pathology is impaired autophagy, Pin1 inactivation, cis P-tau aggregation, cis P-tau-induced endothelial cell damage, and microtubule disassembly [49,96]. 

Just like the liver and kidneys that are affected by preE pathology, the accumulated cis P-tau aggregates in preE patients can spread through the circulation (cistauosis) to brain neurons over time, leading to neurodegeneration and dementia-like features. This hypothesis is supported by the initial report of presence of circulating aggregates of transthyretin in preE patients, and induction of preE-like syndrome in IL-10^-/-^ mice by intraperitoneal injection of preE serum or transthyretin extracted from preE serum [73]. The presence of amyloid-β in addition to transthyretin was subsequently reported in the serum of preE patients regardless of the onset of disease [72]. Interestingly, both hypoxia and serum from preE patients elicit the classical features of preE from human trophoblasts, including increased anti-angiogenic sFlt-1 and sEng, impaired trophoblast endovascular invasion, and defective spiral artery remodeling [49,188]. Additionally, serum cis P-tau is sufficient to elicit all severe preE-like pathological and clinical features in pregnant humanized mice [49]. These defects could be overturned by depleting cis P-tau using stereo-specific mAb [49] in a similar manner observed in other cis P-tau-mediated neurodegenerative diseases [36,41,44,45,126,127]. A similar increase in sFlt-1 and sEng, amongst other features of endothelial damage and preE, was seen in ApoE knockout mice [239], albeit tauopathy was not investigated.

STB stress of preE (stage 1) is common in early-onset/preterm preE and FGR [1,253,258], which is impacted by sterile inflammation and pyroptosis (proinflammatory programmed lytic cell death) [6,268]. Hypoxic stress on placental tissues inhibits the autophagy–lysosomal pathway and inactivates Pin1 leading to the accumulation of toxic cis P-tau amongst other tau protein aggregates, which induces pyroptosis [268]. Hypoxia stimulates excessive ER stress and activation of the unfolded protein response and NOD-like receptor pyrin-containing (NLRP3) inflammasome in primary human trophoblasts [268]. This feature is also seen in stage 1 of preE (STB stress) [253,258]. Although it is not clear if such inflammatory triggers regulate the Pin1-cis P-tau axis in placental tissues, neurodegenerative diseases such as AD also exhibit similar sterile inflammation [269,270]. The ability of pathogenic cis P-tau and other phosphorylated tau aggregates to disrupt the replenishment of the non-replicating STB by the underlying progenitor CTB [271,272,273] may also precipitate STB stress and preE.

While the emerging data discussed in this review appear intriguing, there are more avenues to explore in the role of Pin1-cis P-tau-ApoE axis in the development of preE and its connection to dementia. The current studies predominantly focused on Pin1-cis P-tau axis in placental trophoblast cells, while the presence and function of cis P-tau and other tau epitopes in extraplacental tissues including chorioamnion and maternal decidua, and/or cells in the lower reproductive tract (cervix and vagina), necessitate additional investigation. Moreover, the role of the Pin1-cis P-tau-ApoE axis in preE is yet to be explored. It would also be interesting to determine whether preE-associated tauopathy is mediated by toxic tau proteins conveyed by EVs acting in a paracrine or autocrine manner. Since STBs release EVs when stressed [1], and are known to harbor abundant TFEB [168], expression of TFEB and other autophagy promoters and inhibitors such as ApoE4 in EVs from gestational tissues in relation to the Pin1-cis P-tau axis or cis P-tau levels could be explored. Moreover, since TFEB is the central regulated of the autophagy-lysosome pathway, the effect of the Pin1-cis P-tau-ApoE axis may be secondary to TFEB regulation. Therefore, it is noteworthy to determine whether TFEB regulation affects the Pin1-cis P-tau-ApoE axis directly. The ability of gut microbial SCFAs to modify the influence of ApoE isoforms on neuroinflammation and tau-mediated neurodegeneration can be studied in the context of vaginal microbiota to determine whether a dysregulated vaginal–placental axis can induce preE-associated tauopathy.

## 7. Conclusions

This review highlights the novel role of the Pin1-cis P-tau axis, and to a lesser extent ApoE, in the development of preeclampsia and propagation of cis P-tau-mediated tauopathy from the placenta to cerebral tissues in the long term, leading to dementia similar to other neurodegenerative conditions. Both preeclampsia and dementia are driven by cis P-tau-mediated endothelial dysfunction induced by multifactorial hypoxic stress or injury. The damaged endothelium reduces blood flow, exacerbating ischemia and hypoxia, thereby creating a vicious cycle. Although stereo-specific monoclonal antibody against cis P-tau is currently in clinical trial, and trehalose has shown promise in treating neurodegenerative diseases, further investigation into the Pin1-cis P-tau-ApoE mechanisms is required in other gestational tissues, as well as transcription and soluble pro- and anti-angiogenic factors, and dyslipidemia-inducing genetic polymorphisms that may influence outcomes like preeclampsia and dementia-like features in the immediate and long term.

## Figures and Tables

**Table 1:** Summary of actions of preeclampsia and dementia-associated proteins.

Proteins	Action
Cis P-tau	Disrupts EVT invasion and vascular remodelling [36,44,49]. Increases anti-angiogenic factors [49,76]. Induces tangle formation in neurons [27,28,29]. Induces inflammation [49]. Induces neurodegeneration [36,44].
Transthyretin	Inhibits amyloid-β aggregation and induces neuroprotection [77,78,79,80]. Aggregates form deposits in placental tissue [73,74]. Native form prevents preeclampsia features [73].
Amyloid-β	Form aggregates that damage synapses and lead to cognitive impairment [81,82]. Induces inflammation and vascular damage [81,82,83,84,85].
ApoE2	Reduces amyloid-β [86] and tau [87] deposition in brain tissues. Exhibits anitioxidant and LDL-C lowering activities [88,89].
ApoE3	Generally acts neutral [90,91,92,93]. Ameliorates AD-related increased P-tau and amyloid-β deposition [94,95]. Reduces GABAergic neuron degeneration in cultured human neurons [95].
ApoE4	Impairs autophagy and mitophagy Dysregulates endosomal-lysosomal pathways, enlarged endosomes, and reduced mRNA levels of autophagy markers [96]. Increases tau-induced neurodegeneration, enhances tau phosphorylation, and increases P-tau and tau tangle-like structures [76,87,96,97,98,99,100]. Induces vascular endothelial dysfunction [94,96,101,102].

*AD*, Alzheimer’s disease; *ApoE*, apolipoprotein E; *EVT*, extravillous trophoblast; *LDL-C*, low-density lipoprotein-cholesterol; *P-tau*, phosphorylated tau protein.
